# Book Review: Metapsychology of the Creative Process: Continuous Novelty as the Ground for Creative Advance

**DOI:** 10.3389/fpsyg.2017.01463

**Published:** 2017-08-28

**Authors:** Robert Prentner

**Affiliations:** Philosophy, ETH Zurich Zurich, Switzerland

**Keywords:** creativity research, process theory, microgenetic theory, psychological mitosis, mental objects, Jason Brown

The aim of the book is to elucidate the nature of the creative process from two perspectives, a scientific one and a metaphysical one. On ca. 200 pages, results from empirical research into mental pathologies are merged with neuroscience and philosophy. The book is not limited to a specific idea in psychology but presents a general proposal for the relation of mind and matter. Jason Brown engages in an empirically grounded form of process philosophy which serves as background for the whole book. The reader should thus be interested in the prospect of process philosophy to serve as an alternative framework to the commonly held computational conception of mind.

With this comes the fact that process philosophy is usually hard to read, full of jargon and highly speculative. However, the author works hard to present his ideas in a clear way. Still, chapters 3 and 4 “Causation” and “Process Theory” might be a little difficult to read. On the other hand, I found the addendum that gives a concise summary of Brown's own “microgenetic theory”—a kind of “process neurophilosophy”—very helpful to understand the main ideas presented in the book.

Jason Brown's background is in neurology and psycho-pathology, and he frequently discusses findings from psychology, linguistics, and biology to illustrate abstract ideas. The author also seems to be well-informed in non-scientific fields like music or poetry which are highly relevant to the study of the creative process. As examples, he discusses Mozart, Goethe and Proust throughout the book. This sets the book apart from other forays into process thought that present a purely philosophical perspective.

As those familiar with process philosophy know, creativity is often regarded as metaphysical category that grounds all of reality. Jason Brown is no exception to this, although he—unlike Alfred North Whitehead, the main proponent of process thought in the 20th century—clearly distinguishes between creativity and novelty: The former belongs to the psychology of the human mind, but it is based on a metaphysical view built around the latter. The brain facilitates the “sampling” of novelty which is a feature of nature. To put it into a slogan: “Heightened novelty is creativity; lessened novelty […] is habit.” (p. 108).

This already hints at the connection between some more general themes from process philosophy and more specific ideas about cognition and consciousness presented in this book. Mental objects are not conceived as results of a mechanism of binding that unifies the perceptions and thoughts of an organism, but they rather stem from a development that leads from a psycho-physical whole to particular thought contents. Elsewhere, he refers to the process of object formation in the mind as “psychological mitosis” (p. 184), illustrating the idea that mental development progresses in constrained division rather than in stepwise accumulation of sensory material. An example that the author provides and which lies close to the book's main theme is the act of creativity itself: In all instances, a general concept, category or “field of experience” is unpacked into a particular expression. The progression is from whole to parts and not the other way around. It usually is triggered by an environmental stimulus but not caused by it.

Importantly, this also tells us something about the relation of mental states to the world. No longer should it be assumed that an organism's mental states veridically represent or are “about” an objective external world. Instead, one should think of mental states as appropriate adaptions that emerge from an endogenous activity that connects perception, cognition and behavior. Jason Brown identifies this activity to be realized in the brain and its oscillatory dynamics. The author thereby often speaks of the “mind/brain,” implying that the neuroscientist's brain and the psychologist's mind are “isomorphic” to each other. This is certainly not meant in the commonly held sense that the brain is the material stratum that realizes a certain neurobiological function that we call mind. Nevertheless, the postulated isomorphism could be challenged on various grounds; and consequently, the understanding of the psycho-physical relation would be in need of a conceptual upgrade.

Jason Brown's “Metapsychology of the Creative Process” is a challenging but rewarding book to read. It resulted from the author's 30+ years of clinical study and philosophical speculation. While not everyone who is looking for an empiricist or popular-science account of creativity research will be satisfied, the book has a lot to offer: It connects with process philosophy and contemporary consciousness studies, and it brings some unusual (potentially revolutionary) ideas into the mix. Furthermore, Jason Brown most often manages to elucidate some rather abstract ideas by drawing from a wide range of empirical material. Much more would need to be said about his treatment of, e.g., memory, the self or the unconscious in order to do justice to the enormous scope of this short book.

I recommend the book to either someone who is familiar with research done in psychology and who looks for a philosophical alternative to the computational conception of mind, or to someone who wishes to expand his knowledge in process philosophy to include modern empirical work. The book is probably less suited for audiences that shy away from ideas that are rooted in the demanding concepts of process thought. All in all, the book presents an intriguing idea of relating the mental and the physical.

## Author contributions

The author confirms being the sole contributor of this work and approved it for publication.

### Conflict of interest statement

The author declares that the research was conducted in the absence of any commercial or financial relationships that could be construed as a potential conflict of interest.

